# Ethical challenges in biomarker research and precision medicine – a qualitative study in dermatology

**DOI:** 10.1186/s12910-025-01258-6

**Published:** 2025-11-18

**Authors:** Marie-Christine Fritzsche, Nora Hangel, Alena Michaela Buyx

**Affiliations:** 1 Institute of History and Ethics in Medicine, TUM School of Medicine and Health, Technical University of Munich, Ismaninger Straße 22, 81675 Munich, Germany; 2 Department of Science, Technology and Society (STS), School of Social Sciences and Technology, Technical University of Munich, Munich, Germany; 3 Leibniz Center for Science and Society (LCSS), Leibniz University Hannover, Hannover, Germany

**Keywords:** Ethics, Harm, Epistemic injustice, Data, Biomarker, Dermatology, Chronic inflammatory skin disease, Qualitative methods, Precision medicine

## Abstract

**Background:**

Over 300 million individuals worldwide live with Atopic Dermatitis and Psoriasis, which are among the most common chronic inflammatory skin diseases. Multimodal biomarkers are currently being developed using large-scale data and artificial intelligence to allow for more targeted prediction and to improve treatment of patients with Atopic Dermatitis/Psoriasis. Although this promises enormous benefits for patients, it comes with critical challenges. This article offers an in-depth analysis of the ethical challenges in research and application of data-driven biomarkers in chronic inflammatory skin disease, which, as recent work shows, has not yet been explored in depth.

**Methods:**

We conducted an interview study with 28 members of the BIOMarkers in Atopic Dermatitis and Psoriasis consortium including multiple stakeholder groups involved in biomarker research and application following the COREQ checklist. The interviews were analysed and interpreted theme-oriented using an updated grounded theory approach.

**Results:**

The interviews revealed interconnected ethical challenges described by a wide range of stakeholders involved in biomarker research. Our analysis identified two broad categories of ethical challenges – disease-related and biomarker-related issues – from which three cross-cutting themes emerged: multiple forms of harm, multiple injustices, and multiple uncertainties. Disease-related challenges include covert psycho-socio-physical dimensions of harm and suffering in Atopic Dermatitis/Psoriasis, quality of life impacts, trial-and-error approaches, and communication and expectation management in clinical practice. Biomarker-related challenges range from big data use with multiple biases in the different data-sets, stratification of patients into subgroups, to invasiveness of diagnostic measures, multiple uncertainties and expectation management in science. This article also provides stakeholder suggestions for mitigating harm associated with Atopic Dermatitis/Psoriasis and biomarker use to inform policy development.

**Conclusions:**

The identification of the many ethical challenges uncovered in the interviews and the nuanced view of harm, intersecting injustices including epistemic injustice, and the multiple uncertainties provide crucial considerations for evaluating the risks and benefits of biomarker research and application in healthcare. These insights should inform policy development for data/AI-driven biomarker use for Atopic Dermatitis/Psoriasis and support research practice, public health interventions, and clinical practice to develop and apply medical innovations that are ethically responsible.

**Supplementary Information:**

The online version contains supplementary material available at 10.1186/s12910-025-01258-6

## Background

Atopic Dermatitis (AD) and Psoriasis (Pso) represent some of the most common chronic inflammatory skin diseases with over 300 million individuals affected globally [1–4]. Both diseases can profoundly diminish patients’ quality of life [5–7]. A major challenge is the heterogeneity of these diseases and current treatments do not work across all patients. Despite many new treatment approaches and improvements in response rates in recent years, there are still significant unmet health needs [8, 9]. Many patients with these heterogeneous diseases do not respond to treatment or show a suboptimal response to current treatments [8, 9] and there is a need, for example, to improve remission of symptoms, prevent damage in early stages or progression to advanced stages [10]. Healthcare systems face critical challenges in their reactive rather than preventive approach to care for AD/Pso. Patients often begin therapy late in their disease progression due to system-wide capacity constraints and access barriers [8, 11, 12] This delay can not only lead to avoidable suffering but also allows disease progression to more severe forms and comorbidities to develop.

Evidence shows that there might be subtypes of AD/Pso or even further disease entities that are currently categorised under AD/Pso [13–15]. Thus, international research groups aim to stratify patients into further subgroups to diagnose disease subtypes and find more targeted/precise treatments. High hopes are invested in data-driven multimodal biomarkers [15–18]. A biomarker is “[a] defined characteristic that is measured as an indicator of normal biological processes, pathogenic processes, or biological responses to an exposure or intervention, including therapeutic interventions” [19].^1^ Biomarkers can include histologic, molecular, physiologic, or radiographic characteristics [19]. Their implementation has been well established across numerous medical disciplines, spanning tumour diseases, infectious conditions, autoimmune diseases and cardiovascular diseases. For AD/Pso, clinical assessments, histology, and photography are routinely used for diagnosis and monitoring. Therapeutic drug monitoring (e.g., measurement of drug levels to determine the optimal dose) is used for some drugs in Pso and recently integrated in guidelines in some countries (e.g., [22]). Severity scores are used to quantify the clinical assessments. However, there are no biomarkers for AD/Pso in routine clinical practice. While immunologic biomarkers show significant promise for personalising both disease classification and treatment approaches in dermatology, no standardised or widely adopted methods currently exist in clinical practice [23]. Researchers in BIOMarkers in Atopic Dermatitis and Psoriasis (BIOMAP), a major interdisciplinary European research, have searched for biomarkers with a broad remit – covering different aspects of disease risk prediction, disease progression and severity including clinical, biochemical and genetic markers [16, 24–27].

The consortium has consolidated molecular and clinical data from epidemiological research, disease registries, patient databases and clinical studies, referencing defined outcomes and clinical phenotypes for AD/Pso [25]. Through this collection of multiple data types, biomarker identification and validation are expected to be accelerated [26]. However, prior to introduction the data-driven biomarkers require replication, validation, and establishing clinical utility, acceptability and affordability. The present study therefore brings together the cutting edge perspectives of experts in biomarker research on the use of omics and health data as well as molecular analytic tools and artificial intelligence (AI) to identify or develop these biomarkers for AD/Pso.

In summary, the use of data- and AI-driven biomarkers for diagnostic, prognostic, and therapeutic purposes aims to meet unresolved health needs of patients with AD/Pso offering the potential to enhance disease understanding, earlier and more precise diagnosis, an improved response rate to medication, and better therapeutic control. However, at the same time, such developments may lead to unintended harms. In a Delphi survey, consortium members ranked seven requirements for future biomarkers for AD/Pso as high priority, including performance criteria such as clinical validity, reliability, relevance, and high positive predictive value, clinical purposes such as disease progression and therapeutic response prediction, and addressing insufficient independent validation as an obstacle to overcome [28]. In order to deliver the best possible treatments, the ethical implications of using multimodal biomarkers based on large amounts of data and AI, and the potential harms this may directly or indirectly lead to need to be explored and weighed against the benefits. This is particularly relevant since there is a lack of public awareness about the harm and suffering that chronic inflammatory skin diseases can cause [29, 30]. *Primum non nocere* (first, doing no harm), is a core principle of medical ethics. This applies to the conditions under which both research and clinical application of data-driven biomarkers occur. Our study is on the ethical challenges in data-driven biomarker research and application, focusing on the (potential) harms and not on the benefits, because the immense potential benefits of the use for clinical and public health settings have already been widely reported [15, 16, 24].

Ethical issues surrounding biomarker research and application have been explored, e.g., for psychiatric and neurological disorders (e.g., [31, 32] as well as oncological diseases (e.g., [33]). The aim of this study is to add empirical insights regarding ethical issues that are associated with current research and applications of biomarkers for chronic inflammatory diseases and precision medicine, which, to our knowledge, have not been described in this form before. Thus, we aim to address gaps identified in a recent systematic review of ethical issues in research and healthcare application of biomarkers in the literature [34]. While there are many ethical challenges that we touch upon, a main cross-cutting issue to emerge from the interviews was the question of harm.

Against an overall positive and promising background, we wish to emphasise that the (potential) harms also need to be considered, in a nuanced way, namely by looking at the harms of the existing situation and those of the approaches that aim to improve the existing situation. These have to then be balanced against the immense potential benefits. Ideally, these benefits would be enhanced by avoiding and minimising the harms and further ethical issues so that they outweigh them.

## Methods

### Theoretical background and the interviewers’ position

In order to capture the perspectives of the relevant stakeholders involved in biomarker research, we conducted a qualitative expert interview study [35, 36]. The ethical challenges and the harm they described are discussed against insights from the literature and combined with further theoretical and ethical reflections.

Non-maleficence and beneficence along with autonomy and justice are the four principles [37] that have dominated biomedical ethical analysis in recent decades; harm is a cross-cutting category which is relevant for all four categories. Generally, it plays an important role in moral and legal reasoning (e.g., legal sanctions, [38]). In medical ethics specifically, the importance of having a clear idea of what harm means is obvious. However, the concept of harm (in medical ethics) and its moral significance is highly contested [37, 39, 40]. It has been studied in some philosophical depth in the philosophical and jurisprudential literature [38, 41–43]. The exploration of harm can be divided into two key questions. The first examines what it means to experience harm – specifically, what conditions or circumstances must be present to say that someone has been harmed. The second question looks at the act of harming – what conditions need to be met to conclude that one person has harmed another. When the first question is examined, well-being is essentially investigated and at which point a negative impact on someone’s well-being qualifies as harm. The second question delves into responsibility – attempting to establish the criteria for when someone bears responsibility for causing harm to another. For this article, the first question is of particular relevance.

A very broad account of harm considers it as anything which is in some sense bad for a person. Similarly, benefit would encompass everything which is somehow good for a person. A contemporary common sense perspective takes being worse off as a harm [44, 45]. This typically includes the criterion of well-being as a baseline (worse-off with regard to well-being) and whether someone is worse off compared to this baseline [46]. The main evaluative baselines relate to comparative accounts such as the counterfactual comparative account [47–49] and the temporal comparative account [38, 50] in contrast to non-comparative accounts [43]. A preponderance of authors appear to favour the counterfactual comparative account, the state/world in which the person would otherwise have been is used as a reference base. In accordance with this account, if a particular state/event^2^ had not occurred, “the subject would have been better off” [40]. Temporal comparative accounts substitute the counterfactual by introducing a temporal reference state that characterises the state of the actor prior to experiencing harm [56]. By contrast, non-comparative accounts consider a person to be harmed if a particular state/world is “non-comparatively bad” for a person. Hybrid accounts are proposed that combine these approaches to harm [56].

The concepts of harm and suffering are often closely related. As with the concept of harm, there have been extensive debates (e.g., [57, 58]) what suffering means, ranging from naturalistic, essentialist (e.g., [59]) and phenomenological perspectives (e.g., [60, 61]). Reflections on suffering can also reveal what conceptions of the person are taken for granted, e.g., whether a dualistic view distinguishing sharply between body and mind is assumed.

What harm means concretely, both in respect of specific conditions, diseases and disorders, and for the stakeholders involved as well as in medical action and use of medical innovations, can be highly context dependent. Our article aims to shed light on the many different ethical issues of biomarker research and application, but also on the specifics of harm and suffering as it relates to AD/Pso and biomarkers based on the responses from the qualitative interview study reported on in the following sections. It is against this background that the results are analysed. Given the complexities of defining harm presented above, we did not take a particular position on the meaning of harm but aimed for an open stance with respect to interviewee responses. We first describe and discuss the interviewees’ various perspectives, offering our interpretations in the Discussion below.

### Qualitative interview study

The methods used in our expert interview study adhere to the COREQ (Consolidated Criteria for Reporting Qualitative Research) reporting guidelines [62]. The interviews were conducted by MCF and NH, and analysed by MCF, NH and AB.

#### Theoretical framework

For the theoretical framework used in this study, we followed the updated grounded theory approach from Charmaz [63] as endorsed by Byrman [64] and Yin [65] and drew on Döringer [35] when conducting problem-centred interviews with experts. The interviews were analysed as narratives of the interviewees’ personal experiences and were group-based and theme-oriented.

#### Participant selection

We conducted an expert interview study within the European research consortium BIOMAP, employing purposive sampling [66]. The methodology and methods described in this article largely follow those described in Hangel et al. [66]. Through our participative approach, we identified experts representing the different work packages of the consortium.

Of the total 35 participants approached by e-mail, 28 consented and participated in an interview (80%; 13 female, 15 male). Information about the study was sent with confirmation of the interview date and time.

In addition to the scientific experts in BIOMAP (early career scientists, senior researchers and data analysts), organisation stakeholders, patient board and pharmaceutical representatives as well as advisory board members (internal and external) were included so as to provide a diverse range of perspectives (Table 1 [66]). A few of our interviewees from all groups revealed to be affected by AD/Pso unprompted and without solicitation. Nonetheless, this personal information was either left out of the analysis or completely anonymised in order to maintain the confidentiality of the interviewees.

**Table 1:** Study participant characteristics [66, Table 1]

Participant characteristics		Number (*N* = 28)	Percentage
Gender	Female	13	46%
	Male	15	54%
Stakeholder	Researcher postdoc	7	25%
	Researcher senior	8	29%
	Pharmaceutical representatives	7	25%
	Patient board representatives	6	21%

The researchers on our ethics team were members of the research consortium BIOMAP, and actively participated in consortium activities and meetings prior, during, as well as following the data collection period. Throughout the pandemic of the coronavirus disease 2019, the researchers captured participant observations during virtual sessions [66]. These observations enhanced our comprehension of organisational dynamics within the consortium. Through this approach we gained comprehensive insights into the specific contexts, which enhanced the interpretative validity. The research team applied the embedded ethics approach [67–69]. The team’s involvement encompassed embedded ethics activities across different work packages and task forces, the attendance at analysis meetings of these research groups, patient involvement, communication group engagement, as well as discussion and presentation of research results related to the consortium including stakeholders from different work packages and patient representatives. The initial study results were introduced at an open consortium meeting with BIOMAP members, where open discussions took place. Subsequently, stakeholders from the consortium, including patient advocates, provided input during an internal review session. In addition, we actively participated in sessions focussing on harmonising concepts and variables for the biomarker research in the consortium, establishing a common base for interdisciplinary utilisation of datasets and leading to a peer-reviewed publication [25]. We also contributed to the consortium-directed Delphi study [28]. Furthermore, interdisciplinary scientific papers were written together with researchers from different work packages e.g., [26]. We also contributed to developing further the methodology of embedded ethics in AI in healthcare including a publication with a toolbox for embedding ethics [69].

#### Data collection

We developed a semi-structured topic guide. We presented and discussed this topic guide at an institute meeting with 14 researchers from our institute, who provided feedback as an extended audience beyond our core research team. To pre-test and refine the topic guide, we conducted two trial interviews, which were not part of the subsequent analysis. Questions covered the potential most favorable and worst outcomes from biomarker research, anticipating possible harms related to stratification by patient subgroups and treatment access, socio-environmental determinants and their role, as well as considerations of potential changes in perceptions of these conditions. The interview questions were open-endend and gave the participants the freedom to share anything they found relevant.

Interviews were carried out virtually by the first and second authors from May to July 2021 as well as another interview during September 2021, utilising online GDPR compliant tools. The interviewers collected verbal informed consent prior to the interview taking place. All the interviews were conducted in English with the exception of one in German. The majority of interviews lasted around 50 min, spanning between 17 and 115 min. The interview transcripts were anonymised and double verified by the two interviewers for accuracy.

#### Data analysis

The interviewers first separately analysed and coded two interviews in order to establish the major codes according to themes. Subsequently, they compared the two interviews, refining the codes prior to the three researchers (both interviewers and one research student assistant) coding each interview. For interrater reliability, the complete set of interviews received no fewer than two iterative codings by two to three coders. The anonymised transcripts were analysed using Atlas.ti.

We initially conducted narrative analyses for the identification of the major themes. By analysing all transcripts iteratively, the research team identified the major themes. To validate our findings, we conducted an iterative phase of group-based analysis that revealed additional insights about the major themes. Cross-sectional analysis was conducted for selected aspects. The analysis process was supported by written memos, which all researchers reviewed. The major themes were identified by inductive and deductive, thematic analysis. Codes and key analytical categories were developed through the topic guide (content-analysis) alongside a constant-comparison approach (GT). The interviewers iteratively expanded their codes through discussion while frequently returning to the interviews in order to remain grounded within the empirical data. To analyse the focus topic of this article, ethical challenges and multiple harms around AD/Pso and research/application of biomarkers for AD/Pso in more depth, the code “el_harm_invasive_quality-of-life” was evaluated for the different stakeholder groups separately, i.e., for researchers-postdoc, researchers-senior, pharmaceutical representatives, patient representatives, external consultants and clinicians. The analysis results of the code “el_harm_invasive_quality-of-life” were then compared for the individual interviewee groups and corresponding memos were written. In addition, the overlapping of the code “el_harm_invasive_quality-of-life” with other codes (COOC) in Atlas.ti was analysed. The responses to the question on harm from the topic guide for the interview study were analysed: “What ideas come to mind to mitigate potential harms that arise in the long term, e.g., in relation to stratification of patient groups and the use of Big Data?”, without offering any definition of harm to the interviewees. In addition, prompts (1) and (2) of question 6 were analysed: "What would you consider tacit information at this stage that could become relevant for patients in the future?" What could be the practical implications of disease taxonomies?" In addition, the code “el_policy_guidelines_in.group.procedures” and its COOC with the other ethics codes “el_harm_invasive_quality-of-life”, “el_diversity”, “el_barriers_compliance_IC + general.ethics” and “el_care_prioritisation.of_severity_predispositions” were compared. The COOC of these codes with the code harm was also analysed. To ensure interrater reliability, the notes on these analyses and the interpretation of the results from the first author of this article were reviewed with the other interviewer. Finally, a draft of the article was disseminated throughout the consortium for feedback.

## Results

In this section, we report in detail on the results of the interview study on ethical challenges of multimodal biomarker research, its translation and application for AD/Pso, and the multiple aspects of associated harms, injustices and uncertainties.

Initially, we identified two broad categories of ethical challenges:

1. Ethical challenges associated with AD/Pso as diseases and corresponding medical interventions
2. Ethical challenges in biomarker research and the application of multimodal biomarkers for AD/Pso

**Fig. 1:**
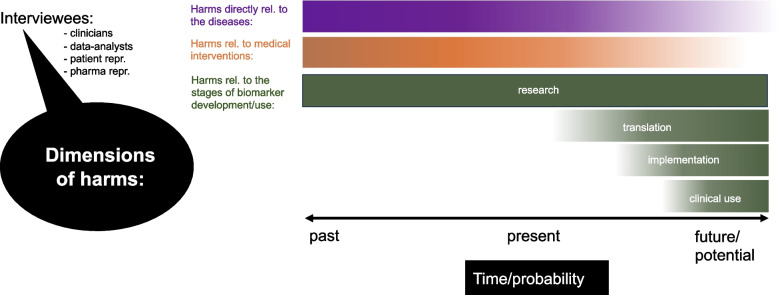
Different dimensions of harm in biomarker research and application

Multifaceted harm [66] is cutting across many different ethical challenges. These are often interconnected and incorporate different *dimensions* of harm. They include time/probability (past, present, and future/potential harm) and different stages of biomarker use for AD/Pso (research, translation, implementation, application) (see Fig. 1). The interviewees, including researchers, patient representatives, clinicians and pharmaceutical representatives, mentioned harm in biomarker research and application both from their own perspectives and those of other groups of stakeholders. The main themes of the multiple harms associated with AD/Pso, medical interventions and biomarker research/application for AD/Pso are mapped in Figure S1 in the Supplementary Materials. Later three themes emerged that cross the two broad categories and the main themes of the interviews: multiple harms, multiple injustices and multiple uncertainties. We discuss those in the Discussion section.

## Ethical challenges associated with AD/Pso and corresponding medical interventions

The interviewees highlighted a spectrum of ethical challenges associated with AD/Pso encompassing stigmatisation, injustices, and particularly multifaceted forms of harm. The harm and risks associated with AD/Pso and corresponding medical interventions described cover psycho-socio-physical dimensions of harm as well as reduced quality of life. The trial-and-error approach was also a key issue as was expectation management in clinical practice, which were described as being associated with harms. The trial-and-error approach in medicine encompasses sequential testing of different treatments until one is found that proves effective for a specific patient. This approach is still used in various clinical situations: for example, in AD or Pso, when a patient does not respond to a treatment. The interviewees expressed the hope that the harms described may be reduced or avoided through the use of biomarkers and could lead to an improved understanding of the diseases and better response rates with more targeted diagnoses and treatments.

### Psycho-socio-physical dimensions of harm and reduced quality of life in AD/Pso

#### Psycho-socio-physical dimensions of harm and suffering in AD/Pso

Psychological harm for patients and relatives/families was discussed on multiple levels by all interviewee groups. The psychological issues associated with the diseases were various, covering individual, collective and cross-generational psychological aspects. A patient representative and a researcher explained that it can be very difficult to cope with the diseases for the patients and pointed to the “detrimental mental health effect” (P01) of AD and the “very heavy mental burden” (P02) of a skin disease.

Interviewees described different forms of fear and anxiety for patients with AD/Pso. A pharmaceutical representative (bioinformatic) talked of “years of fear” (P03). Further psychological harm was described as being connected with multiple uncertainties, which interviewees associated with anxiety, fear and discomfort.

As a topic of particular psychological and ethical relevance with individual and cross-generational impact, the psychological effects of inheritance for people affected by AD were emphasised. One patient representative highlighted how difficult family planning for AD patients could be, particularly when deliberating whether to have children who might also experience harm due to AD. In this context, interviewees addressed concerns about responsibility and empathy for future generations and potential harm that might affect them. Interviewees described frustration and hopelessness experienced by families due to the chronicity and frequent non-response to treatment. Many of the interviewees saw biomarker research as offering real hope in this regard. They suggested that diagnostic and therapeutic biomarkers could help identify suitable treatments for specific subtypes of AD in advance, potentially leading to earlier treatment responses. This would avoid the disappointment and frustration related to the trial-and-error approach, while importantly reducing symptoms early in the disease course. Furthermore, the interviewees described hope that this could prevent certain psychological complications of the chronic diseases and multi-morbidities.

Overall, the harm and suffering associated with psycho-social aspects of AD/Pso were frequently pointed out in terms of isolation, ignorance, non-acknowledgement and stigmatisation. The interviewees highlighted the social dimension of AD/Pso and explained that the consequences of AD/Pso “are very rarely life threatening but they are social” (P04). Isolation and feeling left alone for patients with AD was a recurring theme raised by interviewees. A patient board representative emphasised that AD is a wide-ranging disease of varying severity, which contributes to loneliness as sufferers often either do not have a peer group or if they have a milder form of the disease, their suffering might even be downplayed comparing their type of the disease with severe forms of the disease. They described a vicious circle about hiding the disease, which contributes to the lack of awareness of how wide-spread the diseases are and further increases lack of societal acknowledgement. Interviewees (from all groups) repeatedly referred to a lack of awareness of the disease, of the consequences, its impact and comorbidities.

People are not aware that actually psoriasis has a lot of other conflicts and other comorbidities that come associated with it. And because there is not this knowledge in the population, and we also don't understand the subtypes of the disease then it's more difficult to teach people to understand the people with this disease (P02).

Harm to patients through a sense of social exclusion and a lack of acceptance of the disease were mentioned. Harm through lack of knowledge and understanding of the disease origin and its triggers among the public and general prejudice was described again and again, e.g., when skin diseases like AD were sometimes even considered to be infectious. Thus, stigmatisation was also connected with blaming the sufferers as personally responsible for the disease, or as a pharmaceutical representative (with contact to patients and relatives/family of patients) put it: “the person’s fault” (P04). One interviewee described the shame of patients with AD/Pso: “People do not want to show their skin, people feel that they are being observed, that they're being pointed out.” (P02).

Some interviewees argued that the consequences of the discrimination described and neglect of patients with mild to moderate forms of the diseases would cause even more psycho-social harm. There was a differentiation of harm made by interviewees in respect to severity scales. While the severity scales of AD should yield information on the burden of symptoms, one interviewee mentioned that the assignment of a certain severity classification of the disease was perceived as a judgement from outside, which neglected the life-long suffering that people with mild and moderate eczema can face. This neglect of certain patient groups with a lower severity of the disease was considered to be a form of patient discrimination. The more complex appearance of different types of discrimination described by interviewees is covered in the section on ethical implications of stratification of patients into subgroups. Senior researchers and a pharmaceutical representative referred to further harm for patients with AD/Pso produced by invasiveness of diagnostic interventions in clinical practice and research, using skin biopsies and blood sampling.

#### Quality of life

In terms of quality of life, harm was mentioned mainly at the individual level of the patients. They described suffering harm due to the symptoms of AD/Pso and the comorbidities. The well-known “burden of itch” (P05) was highlighted. It was referred to the physical damage because of itching, “the soreness of the skin” (P05) and the associated sleeplessness at night. Specifically, interviewees with patient contact mentioned that avoiding “itch” should be a stronger priority for research aims.

The interviewees described the ethical implications of suffering harm due to AD and diminished quality of life from a holistic view: it was a life-long disease, and thereby a special form of harm, which impacts the whole body and the “whole quality of life” (P01).

Altogether, interviewees pointed out that harm was caused when patients’ needs and wishes were not fully considered/not properly respected. They suggested mitigating harm by just focusing on the patients’ needs and wishes, considering that the interests and needs of clinicians and patients could be different.

### Trial-and-error approaches

Present and past harms were also described in association with current treatment for AD/Pso. Specifically, if it is not yet personalised with biomarkers. In this context, interviewees repeatedly referred directly or indirectly to past and present trial-and-error approaches to finding an appropriate therapy for patients with AD/Pso. They described some of the negative consequences across different stakeholder groups. Researchers who have had direct contact with patients from personal experience (as opposed to researchers who have not had patient contact) repeatedly mentioned “the psychological burden” (P06) associated with these trial-and-error approaches. The severe emotional impact through these trial-and-error approaches was most often mentioned. One interviewee even talked of an emotional roller coaster and the associated expectation management:

ultimately, what I would like is that there's not a roller coaster of emotions involved in treating patients, the raising of expectations and hopes that this treatment will work, and then they come crashing back down, because it hasn't worked and their expectations raised again, because we'll try this one, then now, and then that fails, or they have secondary side effects. (P07)

Furthermore, interviewees pointed to a waste of resources and time due to the trial-and-error approach. Overall, the trial-and-error approach is contrasted with the hopes for precision medicine with stratification of patients, which could reduce the possibility of multi-morbidities and wasting time testing ineffective treatments.

### Communication and expectation management in clinical practice

Harm from setting false expectations was repeatedly mentioned in the interviews, both in predicting the course of the disease as well as short-term outcomes after new treatments and the trial-and-error approach. Harm to patients, to children and their families and the public due to false expectation management from healthcare providers was mentioned in this regard. “Setting (false) expectations with the public and families who suffer with eczema and psoriasis definitely is harmful” (P01). A patient representative described false predictions for children and their families based on lack of knowledge, which would lead to high and misleading hopes. Misleading messages in false expectation management in medicine were considered damaging: “there is more harm done because people are just saying you might grow out of it (the disease) or not, we don’t know” (P01). The interviewee even talked of myths about the prognosis for AD being spread, which fuelled unrealistic expectations. According to the interviewee, these myths would extend beyond the belief that children will simply outgrow the disease, to include false assumptions that symptoms inevitably improve in later life – after 50 or 60 years—or that certain treatments will always provide a complete cure.

Ethical issues around communication and damaging information were specifically detected in different contradictory statements from healthcare providers and “around all kinds of statements […], all kinds of messages you get from healthcare providers on […] AD and on the effect of human health” (P08). Interviewees considered messages to be harmful that gave reason for them waiting for something that did not then happen – one component of the harm in the expectation and unfulfilled hope was wasting time because of non-response and even more time in terms of harm related to suffering the symptoms of the diseases.

Emotional harm can result from raising high expectation and the negative emotions associated with the expectations not being fulfilled. In summary, the different types and aspects of harms associated with the diseases and their treatments illustrate many of the remaining health burdens and the need to develop better care and treatment.

## Ethical challenges in biomarker research and the application of multimodal biomarkers for AD/Pso

We first addressed ethical challenges associated with the disease and treatments and turn now to identifying potential ethical challenges of biomarker research. Taking into account both should facilitate future ethical evaluations of data-/AI-driven biomarker research and application for these conditions. Even though the expected promises for the application of multimodal biomarkers based on large amounts of data are hopeful and the potential benefits are seen to be enormous, it is also necessary to look at what ethical problems and (potential) harms are associated with this research and application of biomarkers. Our study points to anticipating possible problems to mitigate the harms and to help design research in such a way that it is as patient-oriented as possible.

The main themes associated with the ethical challenges, harms and risks related to biomarker research and application are: stratification of patients into subgroups in biomarker research/application, big data use, knowledge and power, invasiveness of diagnostic measures, uncertainties and expectation management in biomarker research.

### Stratification in biomarker research and application

Interviewees from all interview groups but specifically senior researchers and pharmaceutical representatives described ethical issues and harm directly related to stratification of AD/Pso patients into subgroups using biomarkers. These issues related to multiple forms of inequalities and biases. They referred to distributive injustice as well as implicitly to further injustices such as epistemic injustices without naming them as such. Specifically, they articulated the risk of “favourite subtypes, or the subtypes that are easier to treat” (P09). Potential harm might result for those who were not stratified into a subgroup likely to receive successful treatment. One interviewee anticipated potential harm for a “particular patient subgroup” that “may be very difficult to treat” (P05). “[T]hey [the patients] may be dismissed” (P05). Others expressed the risk of mis-stratification, if patients were distributed into the wrong subgroup and thus would receive the wrong treatment.

Another anticipated risk of stratifying patients into subgroups concerned inappropriate diagnostics for financial reasons and lack of access for certain countries. Interviewees saw a potential risk that certain countries would not have access to diagnostics or treatment based on biomarkers for AD/Pso. In this respect, inequalities concerning clinical trials, which were only very rarely conducted in Africa, were mentioned. One interviewee explained that there was a risk that patients would receive “old-fashioned” treatment, or unsuitable treatment with no improvement in quality of life if there was no access to personalised treatment. Here again, the topic of harm due to discrimination was touched on. Interviewees pointed to the need to ensure fair access worldwide and reach a “(f)air pricing level” (P10).

Higher prices for personalised medicine were identified as a core problem and barrier for equal treatment. Interviewees mentioned the high costs of implementation of biomarkers warning of price orientation with stratification based on risk. In this context, different insurance policies and the respective dependence on local healthcare systems were mentioned. The importance of adequate regulations in the particular political systems, public health policies and structure of the healthcare systems was highlighted.

Apart from inequalities in access to diagnostics and treatments, multiple biases in data for biomarker research were described, most prominently population and selection bias [66]. Interviewees called for the involvement of patient groups “representative of the general population” (P11). They emphasised the need for representation of not only of ethnic background, but also socioeconomic status, explaining that it is mainly middle-class patients who take part in research studies. Furthermore, they highlighted the need to better involve underrepresented or disadvantaged groups.

One senior researcher stressed the real-world inequalities and the importance of considering what was done *after* stratifying patients into subgroups, thereby highlighting the purposes for which data were used.

(I)t's not about the, you know, what we do with the data, it's more about, like, what happens to what we do afterwards. So, the differences and inequalities are there. If we use them to level them up and make everybody's life better, it's good. If we use them simply to label individuals, then this is not okay. (P12)

As a further harm mitigation strategy, the interviewees emphasised the importance of evidencing biomarkers and stratification reliably and with external validity.

### Big data use, knowledge and power in biomarker research

The ethical issues in recent multimodal biomarker research for AD/Pso described in the interviews often related to the use of big data, knowledge, and the power structures in biomarker research. The interviewees pointed to data protection and privacy issues [66], as well as consent issues in light of increased incidental findings with AI and big data use. They also raised ethical issues of trust in data-driven biomarker research and implicitly addressed questions about study participants’ autonomy in the context of AI use for biomarker development. Interviewees of all stakeholder groups explained that multiple potential harms were connected with personal information if it was used in biomarker research and clinical application for AD/Pso using big data and advanced analytics including machine learning (ML) – among other things, harm due to missing data, information and knowledge as well as the misuse of data [66], information and knowledge. Some highlighted potential harm in the use of genetic information and gathering information about personal habits, lifestyle and psychological aspects in biomarker research.

#### Re-identification of individuals

For some interviewees, possible re-identification of a person was considered one of the main sources of harm. For some of the interviewed data analysts, it was the only harm in biomarker research. Especially if big data use combining clinical data with epidemiological and genetic data as a further source of harm for patients with the assumption that “(r)e-identification (was) possible” (P13), it was also expressed as a negative risk for individual confidentiality. Even those who did not see much potential harm in biomarker research and use with ML, and stratification of patients considered re-identification of a person as a source of potential harm for patients and research participants in medical studies.

One interviewee explained that data from Pso patients could also be “very personal data” (P06) even if one could not identify the name, because individual experiences such as trauma were associated with the disease.

Whereas, when you have asked somebody for very personal information and for a lot of people with psoriasis, they have a level of trauma of coming to terms with the disease, so, then that data even though there may not be you know, their name and address on there, it is still very personal data. So, it does need appropriate handling (P06).

One pharmaceutical representative expressed the need for a layer which can ensure de-identification “given the complexity of connecting genetic data, clinical data, [and] epidemiological data” (P13). This interviewee also pointed to fears related to data privacy and security, combined with “reluctance in sharing data” (P13) in “the beginning of the BIOMAP project” (P13), which had resulted in a delay for the progression of the research.

#### Misuse and abuse of patient information

A majority of interviewees expressed worries about the potential misuse and abuse of patient information in biomarker research. In this context, interviewees referred to the potentially harmful use of large amounts of data by insurers if patients are denied insurance cover due to particular traits.

“there is a huge potential of misuse [...] excluding patients from getting an insurance, because they have a specific trait, or just covering less insurance cases, or just treating patients differently, depending on how their body is constituted. Just given on information, which in the future could be available and for this reason, we also try to make it safe and not accessible. And-. Yeah. And I'm actually not sure whether solutions we now have are really guaranteeing this”. (P14)

In addition, the interviewees described potential epistemic harm related to accessing personal data as well as who would have knowledge about future risks. Pharmaceutical representatives advocated the protection of patient privacy by opposing biomarker diagnostic tests in the absence of treatment. In this vein, one of these interviewees made suggestions regarding prohibiting insurers from asking questions related to the risk of developing the diseases, referring to the risk of increased insurance fees and mortgage interest rates [66].

Overall, researchers and pharmaceutical representatives discussed how to use data-driven biomarker research optimally without compromising patient privacy. The interviews reveal different perspectives on enabling research, avoiding current and future harm to patients and other stakeholders, and the potential benefits of data-driven biomarker use in AD/Pso.

#### Incidental findings in data-driven biomarker research

There was a wide spectrum of opinions about the benefits and risks of incidental or secondary findings, i.e., unexpected findings or findings not directly related to the objective of the study. In the topic guide we directly asked about how the interviewees would deal with incidental findings and left it to them to define the term and its context. Answers ranged from ascribing high importance for patients as well as for scientists “often lead[ing] to great discoveries” (P05) to irritation about what to do with them. Some interviewees of the pharmaceutical representatives’ group were more hesitant in judging whether incidental findings were good or bad, mentioning that thorough investigation of medical background was necessary to effectively assess the risk or potential harm of incidental findings to future patients.

In-group procedures for incidental findings and patient consent for incidental findings were considered particularly important in current biomarker research with big data and AI as tools to prevent further harms associated with disclosure of incidental findings. By asking patients if they wished to be informed of incidental findings concerning their health, patients could opt in or out of being told about additional health-related issues. Some patient representatives stated that patients would only want to know if there was an available cure. In addition, the need to inform patients about “the potential reuse of data” (P03) for further research was highlighted. From a researcher's perspective, they argued that in-group procedures for incidental findings should encompass publicising and circulating them in the consortium to benefit interdisciplinary collaboration.

#### Importance of trust in big data research and harm

Public mistrust due to bad data governance was identified as a risk to the progress of biomarker research with data-driven approaches and the use of AI. It was considered harmful for progressing research, also due to the slowing down effect it might have on the development of treatments. The risk of losing support regarding big data initiatives was articulated along with the risk of losing the opportunity to do such research. Here, the tension between the goal of precision medicine to help patients and the (risk of) misuse of patient information becomes apparent again. Overall, the stated need to “act with high integrity and stewardship towards […] data” (P15) can also be interpreted as a way to secure trust.

#### Lack of knowledge about the disease and research vs. empowerment

Primarily participants from the patients' board related psychological harm with anxiety and stress to insufficient self-efficacy and a lack of patient empowerment. Researchers with patient contact also reported that patients who agreed to take part in medical studies often did not understand the medical content in the informed consent process. Among other things, this might cause harm through false expectations that could not be met.

Knowledge about the diseases and research was seen as potentially dis-/empowering by many interviewees from the various stakeholder groups. Some mentioned that knowing more about the diseases and research would enable patients and families to adjust their lifestyle. They even pointed to the positive influence of gaining more insights from research studies, even if these results did not yet offer new treatment options. Instead, they repeatedly emphasised the importance of patient-oriented research outcomes. Hence, patient representatives presented themselves as facilitators helping to communicate and connect families affected by AD/Pso. One patient representative named charities inform patient groups and their families of new research developments in lay language.

Better understanding of the diseases and the mechanisms in research - key word transparency - was seen to increase confidence for patients and was believed to have an empowering effect for patients, patients' families and other stakeholders. In particular, interviewees with patient contact highlighted the need to raise public awareness of what patients with AD/Pso go through in terms of what influence the disease has on their whole life, treatment costs and the sometimes frustrating journey to find a suitable treatment. Comorbidity problems associated with the diseases are not widely known nor what triggers the diseases. They called for a better general education on the influence of environmental factors on disease progression.

### Invasiveness of diagnostic measures for biomarker use in AD/Pso

Senior researchers and a pharmaceutical representative referred to harm for patients with AD/Pso produced by invasiveness of diagnostic interventions in research using skin biopsies and blood sampling. In addition, one interviewee pointed out the particular harm associated with the invasiveness of skin biopsies for patients when specific areas of the body were affected with the disease – “especially when the skin on the face or in a joint is involved” (P05).

One of the senior researchers raised ethical questions related to the invasiveness of procedures in science: “is that ethically justified to perform invasive procedures on patients for the sake of scientific progress?” (P11). One pharmaceutical representative cautioned about doing “science for science’s sake” (P05). Invasive procedures that benefit research and possibly future patients – but not necessarily and often not directly the patient donating tissue—were questioned. The interviewees generally highlighted directly and indirectly different interests of patients and clinicians, which had to be considered in decisions about whether invasive diagnostic tools for AD/Pso were justified. The need for alternative methods which are less invasive was expressed. In summary, many interviewees pointed to the need to avoid invasive interventions whenever possible in order to mitigate harm associated with biomarker research.

Overall, junior researchers tended to see less harm in biomarker research for AD/Pso, including invasive techniques such as blood sampling and skin biopsies. Those who did not see much potential harm regarding the invasiveness of diagnostic measures nevertheless emphasised the identification of a person as a possible source of harm. As long as the identification of persons was not possible, they envisaged no bigger potential harm.

### Multiple uncertainties in biomarker research and application

In the interviews, a prominent topic was uncertainty [66]. Different forms of uncertainty connected with the diseases, research and use of biomarkers for AD/Pso were mentioned. These uncertainties stand in contrast to the interviewees’ wishes, hopes and aims for certainty, control and security. In the following, we briefly elaborate on this tension.

Researchers, patient and pharmaceutical representatives (all interviewee groups) expressed the strong wish for certainty and control, using the words “make sure” several times as well as superlatives regarding regulations, procedures, governance, and ethical standards. The need for ethical reflection on policy development was also pointed out by the interviewees. They felt that certainty related to governance frameworks, control, vigilance and safety should be one of the top priorities. The need for governance frameworks concerning data as well as organisational procedures (in general) was considered particularly important. As one interviewee put it: “We should remain vigilant on the use of rigorous accurate procedures” and we “should be absolutely vigilant on how the data are handled” (P18). P18 believed harm resulted from insufficient governance structures and control. Several interviewee groups particularly connected data topics with certainty if it was about protecting patients in large-scale data research.

And we just need to make sure that we, […], are very transparent about how we use the data that we have, and that we are good stewards and have safekeeping of the data in mind. (P15)

Thus, the wish for data safety on several levels was frequently expressed by many interviewee groups – very obviously data security, data protection, safe storage of data, data anonymisation, “safeguard[ing the] individual” (P03) and safeguarding/checking whether the research question is a good one. One interviewee felt all concerned should “live up to the highest ethical standards on data sharing and who is getting access to the data” (P10). Interviewees generally pointed to the need for reassurance regarding appropriate use of personal data. However, statements on wishes and demands such as the ones that it should be ensured that “data are as secure as absolutely possible” (P03) stand in contrast to the uncertainty regarding whether the current solutions “are really guaranteeing” that information is “safe and not accessible” (P14).

Some interviewees saw knowledge as a guarantee of certainty and clarity. Hope for certainty and clarity through stratification of patients into subgroups and subsequent emerging disease taxonomies persisted among the interviewees. However, this also stands in contrast to the many uncertainties related to current biomarker research using large-scale data with ML, and precision medicine. There is uncertainty as to whether biomarker research and precision medicine for AD/Pso delivers what it promises, especially with regard to big data topics with the use of advanced analytics in healthcare such as security of anonymisation.

Several interviewees described uncertainty about ethical and social issues and ethical standards in biomarker research and application as well as internal procedures and regulations and governance structures in biomarker research with large amounts of different health data sets and ML. One interviewee also mentioned uncertainty concerning opinions/attitudes related to harm, “what harm can be done” (P11) in biomarker research and precision medicine with biomarkers. One interviewee referred to the potential harm from participating in experimental trials of new treatments at an early stage, which are more uncertain and could potentially cause more unknown harms. The potential harm is partially mitigated by not including children in studies until a later stage, when trials are considered safer. Yet this generally could create further potential harm for children with the disease as knowledge of new treatments is not gained as quickly for them. Interviewees pointed to further epistemological uncertainties and research ethics problems on multiple levels. Two interviewees elaborated on the problem of understanding the influence of environmental factors on the disease. Another interviewee outlined the difficulty of identifying causes and consequences in biomarker research, describing it as the “chicken-and-egg problem”.

Multiple uncertainties were also covered in relation to communication for research participants and patients – not only regarding expectation management, but also concerning how to communicate uncertainties to the stakeholders involved and to the public.

### Expectation management in biomarker research

Specifically patient board stakeholders criticised extremely positive promises and overly high expectations raised for the outcome of research projects, including the promise that a certain treatment would “solve everything” (P08). It was perceived as a potential source of harm to “publicise or to trigger high expectations about new treatments in the short term” (P01). Furthermore, the interviews pointed to the challenge of managing expectations when there really was a lack of knowledge about the diseases. In some interviews, there was the assumption of an uncertain environment in the current situation for AD/Pso where knowledge about the diseases was lacking, and where knowledge and guidance and even guidelines for patients were needed: “I think that people do like, do actually like guidelines because they give them a sense of knowing what to expect and what is expected.” (P07). According to interviewees, good, realistic expectation management was required to avoid overpromising in science communication. One interviewee even insisted it would be better not to “promise anything” (P08)*.* However, while some interviewees showed a very differentiated view of the multiple harms caused by false expectation management, others revealed very high, unrealistic expectations of BIOMAP in the assumption that diseases could be eliminated soon through the application of biomarkers.

## Mitigating harm strategies and the inclusion of patient perspectives

In addition to the specific mitigating harm strategies suggested by interviewees to address handling large amounts of data, information and knowledge in research and use of biomarkers for AD/Pso, stratification of patients into subgroups, and regulations, interviewees added general mitigating harm strategies focussing on the inclusion of patient perspectives (Table 2). In general, awareness and knowledge in biomarker research of the contexts in which biomarkers are applied were considered critical for the use of complex compound biomarkers, along with the skilled professionals able to understand and implement them.

**Table 2:**
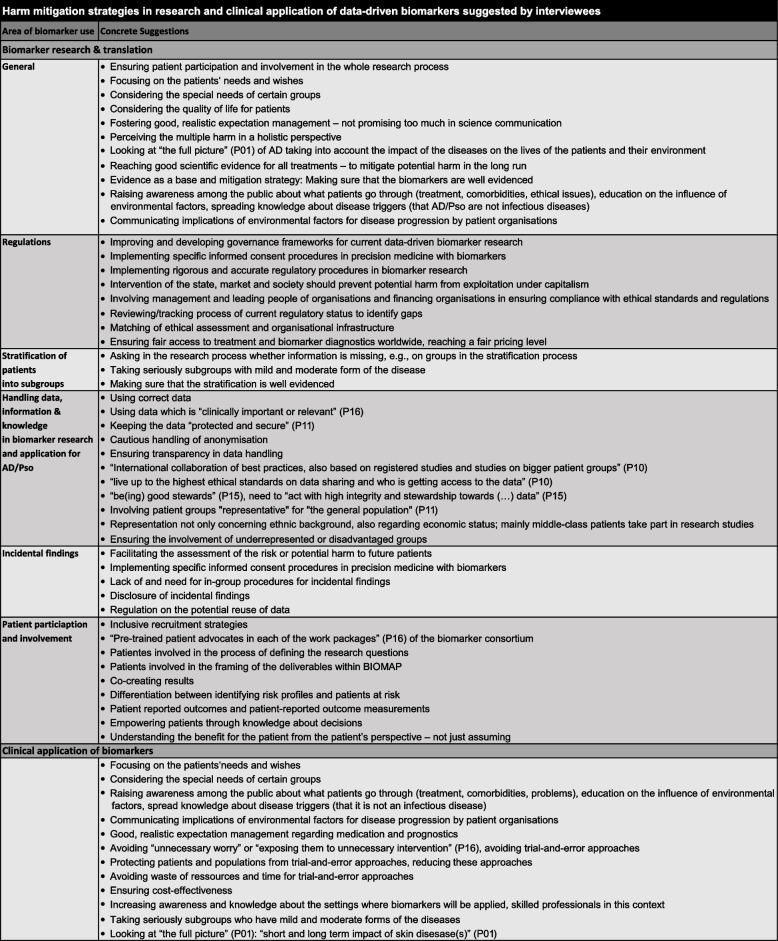
Mitigating harm strategies from the interviewees’ perspective

Several interviewees considered it important to integrate patients and their needs and wishes in the whole research process – even at the beginning when formulating research questions and framing the deliverables in the work packages of international research consortia. One interviewee suggested having pre-trained advocates for patients in each work package of the consortium. On the whole, the interviewees promoted inclusive recruitment strategies. Patient as well as pharmaceutical representatives agreed that medical innovation should be directed towards patient needs. They explained that it was necessary to understand the benefit for the patient from the patient’s perspective and not just by assuming the benefits they may perceive.

Interviewees pointed out that there was also a need to have more patient-reported outcomes measures and to re-evaluate how patient-reported outcomes are assessed. They described the need to use appropriate scales of quality of life that reflect the specific harm as well as the needs of the patients. Evidence and clinical trials that were focused on evaluating the outcomes based on the quality of life were called for. In general, interviewees advocated for patients to be empowered through processes integrating their positions in research decisions.

## Discussion

In our international qualitative interview study on data-driven biomarkers for AD/Pso, we examined ethical challenges in biomarker research and application. Our analysis initially identified two broad categories of ethical challenges: AD/Pso disease-related issues and biomarker/application concerns. From these, three cross-cutting themes emerged: multiple forms of harm, multiple injustices and multiple uncertainties. These themes reveal the importance of not only considering the biomarker technology-related challenges but also the disease-related aspects for ethical analysis.

### Overview

Many ethical issues described by the interviewees reflect debates on precision and personalised medicine [70, 71], and systems medicine [72] across various medical fields. These include increased costs of targeted treatment, resource allocation and inequalities regarding access to healthcare [73, 74] early prediction [75, 76], issues related to a hype around precision medicine, AI and omics data [77], and those around stratification of patients [78]. Biomarkers can be considered as paradigmatic instruments for precision medicine. Several ethical challenges from the interviews reflect discussions on AI and big data in healthcare covering data handling with privacy issues, data protection, and enhanced predictions [79–83]. Recent ethical discussions have extended to healthcare research that uses heterogeneous large-scale data [84]. These datasets encompass multi-omics data with genomics data from Genome-Wide Association Studies (GWAS), molecular, environmental and clinical data, partially analysed using AI – a development in biomarker research that is also present in the research conducted by the consortium of which the interviewees are members.

The results show the importance of considering the ethical aspects and (potential) harms related to biomarker research and use for AD/Pso, and to the disease. This can help to conduct harm-benefit and broader ethical assessments. More precise diagnosis and treatment using biomarkers would not only improve response to therapies, but possibly avoid trial-and-error approaches that can lead to severe psycho-social-physical burdens. In addition, many other harms associated with the diseases might be alleviated if symptoms were reduced. At the same time, it is clear that biomarker research and the application of biomarkers for AD/Pso can be associated with ethical issues that need to be minimised as much as possible to comply, among other things, with the principle of *non nocere* and different forms of justice. The interviewees also suggested various strategies for mitigating harm, which we have summarised above. A recent systematic review found that ethical aspects of biomarker research and application for AD/Pso lack in depth description in the literature [34]. The results of our study go some way to addressing this gap and provide in-depth perspectives on the ethical challenges of data/AI-driven biomarker research and application for AD/Pso, that have, to our knowledge, not been described before. The insights not only enhance our understanding of ethical issues in current data-driven biomarker research for AD/Pso, but also contribute to broader reflections on research and healthcare involving heterogeneous large-scale data and AI.

### Key Themes

#### Multiple harms and suffering associated with AD/Pso and biomarker research/application

The interviews yielded detailed insights from the various stakeholders involved in an international research consortium on biomarkers for AD/Pso. These highlight the high level of harm and suffering for people with chronic inflammatory skin diseases. Common harm and suffering that is referred to elsewhere in the context of AD/Pso includes the reduced quality of life because of itching and (socio-) economic disadvantages due to problems either in or in gaining employment [85–87]. Individual suffering and burdens are illustrated on scales for AD such as the AD, AESEC, DLQI and POEM [29]. The many nuances of the physical and/or psychosocial burden of AD/Pso [29, 88–90] and the tremendous impact on quality of life are evident from the interviews. The interviewee responses also reinforce findings from the previous literature on the burden of the trial-and-error approach to AD [91, 92]. In this context, it is important to highlight that the interviewees had different "direct and indirect" experiences with AD/Pso, which were considered when analysing AD/Pso-related suffering and harm and the use of biomarkers: some had the disease themselves, others had relatives with the disease or were clinicians who treated AD/Pso patients, some were people who had represented the interests of people with AD/Pso for many years, and several interviewees were involved in biomarker research and use in other ways.

The present study offers a springboard for exploring how different conceptual accounts of harm could contribute to medical ethics analyses in various practical settings, from research and development to clinical ethics. The interviewees’ notions of harm often correspond to a comparative counterfactual account of harm (state/world in which the person would otherwise have been is used as a reference base), wherein the three different states of a) having the disease(s), b) undergoing medical interventions, or c) being affected by biomarker research/potential application, and compares these states to one without the disease/medical interventions/biomarker research/application. Thus, the evaluative baseline here is the state in which the person would otherwise have been. In contrast, there are also examples that can be assimilated to the non-comparative account of harm, where a certain state is considered intrinsically harmful as it is solely bad for a person. A few interviewees seemed to use this account when describing, for example, certain psycho-socio-physical harm associated with the disease. The non-comparative account of harm was also used by patient representatives when the diminishment of human flourishing itself was considered as harm. At the same time, it was considered separate from both the patient’s earlier state and the deployment of AI and large-scale data for biomarkers. In their assessment of potential benefits and drawbacks of data-driven biomarkers they considered both the corresponding ethical issues and how the diseases have limited their capacity to flourish over their lifespan – limitations that could potentially be reduced through more precise therapeutic approaches. The interviewees’ different accounts of harm suggest that hybrid accounts of harm [56] could be valuable, as they might help integrate various stakeholder viewpoints and different conceptual frameworks. One potential way forward could be to explicitly clarify how harm is understood before conducting ethical analyses, which might lead to more differentiated outcomes and help prevent misunderstandings.

The non-identity problem [93, 94] is indirectly referred to when the interviewee (patient representative/patient) reflects on possible harm to children who may have AD (genetic association) and the interviewee’s responsibility for that harm. Overall, many interviewees seem to understand suffering as a multidimensional phenomenon that can be described not only by findings from the natural sciences but also from other disciplines (see also [95]) as well as by personal experience (understood as “Lebenserfahrung” and “Erlebnis” [95–97].). For example, according to one interviewee, personal experiences inherent in suffering should be taken into account in privacy issues, e.g., when traumatic experiences associated with the diseases are incorporated in medical data.

Regarding the underlying conceptualisation of a person, many of the interviewees do not start from a mind–body dichotomy, but base it on a psycho-socio-physical understanding, taking into account many other environmental, cultural, and social factors that compound and influence the suffering associated with the diseases. A prominent example of this is when the consequences of AD/Pso are described as “very rarely life-threatening, but social” (P04).

Pursuing evidence-based medicine while considering these different dimensions of suffering does not appear to present a problem theoretically for several interviewees. The translation and implementation of data-driven biomarkers that address the complexities of ethical, social, and environmental factors as well as individual and collective harm and suffering, however, do appear to present an enormous challenge.

The interviews not only highlight the importance of achieving a high level of evidence for data/AI-driven biomarkers and stratification of patients into subgroups, but also empirically inform ethical issues, individual and collective harms, and mitigation. For many of the interviewees, consideration of personal experiences related to the diseases and their associated harms and suffering seems to be as important a component of personalised medicine as biomarker-based stratification.

#### Multiple injustices

The results presented here suggest that different intersecting injustices, intersectional discrimination [98–100] and epistemic harm is a central ethical issue in data-driven research and use of biomarkers for AD/Pso. This includes issues of distributive justice with a lack of access to diagnostics with multimodal biomarkers, more expensive targeted therapies, and population and selection biases associated with the data comprising multi-omics data used to develop biomarkers. There is the well-known historical lack of diversity in genetic studies [101], and although there have been measures to increase diversity (e.g., [102], genomics data from large studies often used for biomarker research (GWAS) are still primarily based on white European populations [101]. Overall, the interviews indicate that multiple biases might arise in biomarker application for AD/Pso including 'race' as well as socioeconomic status. Hence, transparency is needed to show exactly which population data the biomarkers are trained on, which meta-data were used and thus, for which groups the biomarkers can be applied. If data are to be truly representative and accurate, structured measures must be taken to reduce these biases. In light of the different, ambiguous or non-existent use of definitions for categories such as 'race' and ethnicity even in genomics and genetics research [103], an analysis of biases on a meta-level could be valuable. The interviews revealed specific concerns about “favourite subtypes” that are easier to treat, potentially leading to the dismissal of difficult-to-treat patient groups. Additionally, risks of mis-stratification could result in wrong treatments. Furthermore, interviewees point to the issue of potentially high costs for targeted treatments based on data-driven biomarkers, which is a common concern in precision medicine [73] and could increase barriers to treatment.

The interviews underline that in addition to distributive justice, other forms of injustice play an important role for people with AD/Pso, biomarker research, and precision medicine. Several examples are given in the interviews, which show that further issues of social justice and, in particular, epistemic injustice, as described by Fricker (2007), need to be considered. Epistemic injustice manifests when a person is wronged “specifically in their capacity as a knower” [39]. The interviews make this particularly apparent as the topics of how knowledge and power are used are frequently connected to the interviewees’ descriptions of harm, discrimination and injustice – even if there is an effort to invite underrepresented populations to participate in medical studies. The interviews indicated that harm can be caused when the concerns of certain people and groups were not fully considered. The interviewees pointed to the lack of awareness of the individual burden of the disease among the public and healthcare professionals compared to other diseases, as described by Ring et al., 2019. This can lead to testimonial injustice [39] when those concerned are not taken as seriously as others with a similar burden but a different disease or disease subtype – a clear example of testimonial injustice where patients’ credibility is questioned specifically due to prejudices about a specific type of disease, also described for psychiatric disorders [104, 105]. In this vein, several interviews also revealed that currently within the group of AD patients, subgroups noted a lack of recognition of their voice and experiences, such as the severity of the disease they experience, which may not be reflected in disease severity classifications. This again represents testimonial injustice in that certain subgroups’ presentation of symptoms can be given less credibility due to certain disease classification systems. In addition, the interviews show that stratifying patients into subgroups using biomarkers may neglect further groups of people, and it is worth questioning whether some groups whose voice is not equally validated may also be neglected. Again, this might not only be a matter of distributive justice but testimonial and hermeneutic injustice. The latter would apply when subgroups are not created due to “a gap in collective interpretive resources put(ting) someone at an unfair disadvantage when it comes to making sense of their social experiences” [39]. This represents hermeneutic injustice [39] as these groups lack the conceptual resources to understand and articulate certain circumstances and their lived experiences.

Lack of self-development seems to be a harm, which emerges in several interviews. This can particularly concern children and young people, who may not be able to pursue the educational and professional paths they want to due to the diseases. Additionally, as Fricker describes, testimonial injustice can induce harm, which runs deep “in the psychology of a subject” [39] and “can cramp self-development” [39]. There seems to be a risk that AD/Pso and their multiple harms, as well as the lack of treatment or access to diagnosis and treatment, prevent those affected from human flourishing – maybe a certain type of harm on a “higher” level. This is a possible yardstick by which suffering and medical interventions can be co-evaluated [29, 39, 106, 107]. It may also facilitate the more holistic understanding called for by interview participants.

Another prominent issue arising in the interviews was that knowledge production and dissemination are insufficiently critically examined and embedded power structures in data-driven biomarker use are often not acknowledged or discussed. This indicates that further epistemic injustices could occur and need to be addressed by further research, for example, when data/AI-driven biomarkers for diagnostic purposes gain more relevance in defining diseases. Given the potential over-reliance on and opacities of certain AI systems, debates on epistemic injustice become even more relevant. Such research would also foster public debates and the development of the terms for injustices, which may currently be lacking, itself an example of hermeneutic injustice. Further in-depth analyses of these aspects are needed but are beyond the scope of this article.

Although this paper focused on the multifaceted forms of harm that were described in depth during the interviews and are important for differentiated harm-benefit analyses, comprehensive ethical analyses need to extend beyond these aspects to consider other very relevant ethical issues and benefits.

Given the enormous impact that diseases can have on patients' lives, their well-being and flourishing, the need for early diagnosis and better treatment becomes even more apparent—and if the use of data/AI-driven biomarkers could accelerate and improve effective treatments or even early prevention, the need to promote this research and its application becomes even more urgent. However, the conditions under which the research is conducted require continuous reflection and assessment, together with sound standards as the technologies behind data-driven biomarkers advance.

#### Multiple levels of uncertainty

Many interviews reveal a tension between the hopes and wishes for certainty and the many levels of uncertainty around biomarkers and precision medicine. The research undertaken in BIOMAP is directed towards many of the uncertainties inherent in the heterogeneous diseases AD/Pso [66]. One goal of the consortium is to find preventive and predictive biomarkers that reduce uncertainty about the course of the disease, developing comorbidities and response to treatment [24]. As indicated in the interviews, there is still some way to go before many of the biomarkers for AD/Pso can be used for diagnostic, predictive and other purposes. At this stage of research, there are still uncertainties regarding multimodal biomarkers for AD/Pso patient stratification and future personalised treatments. Therefore, many of the statements made by the interviewees were hypothetical, including some statements about potential harms – this also carries inherent uncertainty about the potential harms and ethical issues described. However, this early anticipation of harms is a strategy to avoid certain harms early on and provide more certainty about ethical issues of biomarker use in research and in clinical settings. The advantage of examining the ethical and social implications of scientific and technological developments is that it is preferable to anticipate developments when they are still malleable, even if at this stage many aspects around the application of these biomarkers are still unknown and uncertain [108].

The interview study shows that many uncertainties have to do with the use of large-scale data and ML use. In the future, there might be more diverse health data available and large amounts of data may be handled more swiftly and transparency may be more easily accomplished by more advanced analytics. Meanwhile, the urgent wishes and needs for certainty regarding data governance and the fulfilment of ethical standards in this area to protect patients and research participants have to be taken seriously. At the same time, the interviews have also demonstrated how important it is to solve these problems quickly (as far as possible) so that research progress is not hindered.

Particular uncertainties will probably persist in data-/AI-driven biomarker research and their clinical application. The question remains how much certainty is needed before medical innovations can be applied in clinical practice. As shown, the answer is not only to be found in the scientific insights of biomarker research, but must be part of continuing reflection and wider debate on the ethical issues, harms, risks and benefits of the biomarker innovations involving all stakeholder groups and societies. Even though there may be uncertainties which are more difficult to approach and that have to be part of a long-term agenda, some uncertainties might be easier to handle. These are also detailed in the interviews. They concern, for example, communication and expectation management in science and clinical practice. Expectations play a crucial role in shaping scientific and technological development [109, 110] and can be considered as serving as performative elements in innovation processes [109]. Expectations, visions, hopes, promises, hypes and futuring are closely related in science and technology. The dynamics of very positive expectations, hopes and hypes have been described for biotechnologies, in genetics and precision medicine [77, 111], as well as for the use of big data and AI in healthcare [112, 113]. These are all part of the research object here.

The interviews indicate, in line with recent dermatological literature on antibody treatment for AD [114], harm for patients induced by raising unrealistic hopes. Interviewees cited a loss of trust which they attributed to unrealistic expectations and viewed as a risk to biomarker research. It should be noted, however, that the term “unrealistic” itself warrants careful use in this context, since for technologies still under development, the final form of the product toward which hopes and expectations are directed remains unknown [109].

One strategy to avoid harm due to expectation management may be by professional communication of the uncertainties of research endeavours to patient groups, research participants and the public based on research findings,^3^ while recognising that expectations themselves serve important functions in innovation development [109]. Further ethical dimensions are to be considered in the expectation management of emerging technologies [116], including the larger question of how research can and should be conducted responsibly. Scholars have emphasised that expectations in science and technology frequently reflect strategic efforts to secure funding and backing [110, 117], remain challenging for non-specialists to verify, and typically, they turn out to be partial or false [116, 118]. The insights of this study also call for a debate about the role of honesty, hope, and deception [119] in the context of data/AI-driven biomarkers. The interviews indicate that strategies are necessary to systematically balance hope and more neutral communication of findings. Recent biomarker research (for AD/Pso) with large-scale data can learn from current debates on responsible AI use including those on the lack of explainability or explicability [120–125].

The interviews suggested the need for additional ethical standards together with clear regulations for data/AI-driven biomarker use to be developed for AD/Pso, which cover the ethical issues mentioned. This particularly concerns topics of scientific standards combined with digital ethics for data/AI-driven biomarker research and clinical application. These ethical standards should further develop guidance on the use of large-scale data and AI for biomarkers – specifically regarding the use of representative and diverse data for biomarker research to increase access to diagnostics and treatment rather than create barriers. They may also need to provide additional direction for handling increasing incidental findings and data privacy when anonymisation of data is not possible due to improved AI. Furthermore, standards would offer guidance on how to protect those groups that cannot be stratified into a subgroup when there are increased amounts of biomarkers due to AI in respect of distributive and epistemic injustices. However, these ethical and regulatory frameworks should not function as hurdles in science, but also increase certainty. In summary, the many ethical issues in data-driven biomarker research and development for AD/Pso have demonstrated the importance of embedding ethics in respective research projects [68, 69, 126] and in the development of regulations for biomarker use in AD/Pso. As made clear in the harm reduction strategies suggested by the interviewees, the research and application context of complex compound biomarkers is essential for biomarker evaluation [20] and thus the respective evaluation and ethical analyses must be adapted as well.

On the whole, the study findings show that a holistic view of the multiple interconnected harms, risks and ethical issues surrounding the disease and on the research and use of data-/AI-driven biomarkers is warranted. The early identification of (potential) ethical issues and the nuanced view of harm and suffering can raise awareness and improve ethical analyses. This can help to better inform research and clinical practice, policy development, and public health interventions in order to develop and apply data-/AI-driven biomarkers in healthcare in ways that are ethically and socially responsible.

### Limitations

#### Limitations of the method used for the interview study

This qualitative study does not comprise statistically representative data. While the results cannot be generalised in longitudinal or quantitative terms, they offer valuable insights into interconnected topics in a certain context through our inclusion of a wide variety of experts involved in biomarker research. This study has thus captured key aspects of ethical issues associated with current biomarker research and application for AD/Pso.

In addition, we used lay language for the interviews, as the interviewees had different levels of scientific and medical knowledge. Thus, while no medical details were captured, a common understanding formed the basis for exploring the ethical issues related to biomarker research, which was the purpose of this study. As the focus was to show the perspectives of study participants, detailed theoretical ethical reflections are beyond the scope of this article.

#### Topic gaps in the results of the interview study

Although it is a strength of the study that it reveals multiple perspectives and ethical issues in data-/AI-driven biomarker research and use for AD/Pso, there are a number of topics that could be explored further as well as studies needed to explore them. Our study did not capture all affected groups, including different patient groups. Ideally, the study participants would also comprise e.g., pregnant people, children, young and old people as well as further affected people such as friends and relatives. Future research could explore in-depth injustices, including epistemic and structural injustices, related to AI use for biomarker research and application. This is beyond the scope of this article, which presents an overview of ethical challenges identified through a qualitative interview study, focussing on multifaceted associated harms. While more nuanced understanding from different stakeholder perspectives might help to better weigh harms and benefits, and lead to more differentiated outcomes of ethical analysis, we would like to emphasise the need to consider other ethical issues, both those described here and those that might not yet be addressed. This should also avoid overstretching the concept of harm. For example, and as scholars have emphasised, other ethical issues such as autonomy, also viewed from relational perspectives [127], should not be overlooked in subsequent ethical analyses, particularly in relation to the progressing use of AI and large-scale data and privacy issues.

Another limitation of our study is that many interviewee statements about potential ethical issues and harms related to data-/AI-driven biomarkers were hypothetical. It is important to be aware of these uncertainties and to continuously critically examine them while research advances. We deliberately build upon these uncertainties so that ethical problems are identified and addressed early in the research and development of data/AI-driven biomarker research, thereby averting serious consequences for individuals and society.

#### Outlook for policy development

The interviewees made several suggestions for how the multiple harms could be mitigated (see Results and Table 2). These insights together with the further insights of this and our previous studies [34, 66] can inform policy development not only for the use of data-driven approaches for multimodal biomarker research/use for AD/Pso, but may also be valuable for research and application of complex compound biomarkers for other diseases.

## Conclusions

The in-depth findings of the study provide empirical insights into the ethical challenges of biomarker research and application for chronic inflammatory skin diseases, which is a clear gap in the current literature. Specifically, the study provides a more differentiated and broader view of what harm and suffering can concretely mean in the context of AD/Pso and for data-rich biomarker research and application from various perspectives of stakeholders involved in biomarker research, which enables a more nuanced harm-benefit analysis. The ethical issues and multiple harms were mainly connected to themes such as the psycho-socio-physical impact of the diseases on patients' lives, various uncertainties in treatment and biomarker research, expectation management in research and medical practice, stratification of patients into subgroups, big data use as well as knowledge and power. While the harm associated with AD/Pso may be reduced through improved diagnosis and treatments based on multimodal data/AI-driven biomarkers, the benefits of biomarker use have to be weighed against the ethical implications and potential harms associated with biomarker research and application.

The findings show multiple injustices and discriminations that often intersect and are associated with AD/Pso, as well as current clinical practice and biomarker research with the use of large-scale data and AI. These injustices include e.g., which biases are implemented in the data used for biomarker development, for which populations the biomarkers can be used and how access to biomarker diagnostics and more targeted treatments can be ensured worldwide. Epistemic injustices identified in the interviews relate to the relationship between power and knowledge, especially in big data and ML use in healthcare and in this case for data-driven biomarker research in dermatology. This needs special attention, and can better be understood by including different conceptual lenses, such as that proposed by Fricker (2007), which would help individual groups that otherwise do not have a voice and do not get enough attention to be heard.

The action-based suggestions for mitigating harm strategies by the interviewees highlight comprehensive patient involvement throughout the research process—from defining research questions to including pre-trained patient advocates in research consortia—while ensuring ethical data handling in biomarker research for AD/Pso. The interviewees stress orienting medical biomarker innovations toward patient needs and wishes through patient-reported outcomes, adjusted quality-of-life scales, and patient empowerment in research decisions. The recommendations emphasise the importance of careful patient stratification to ensure all subgroups are considered, while maintaining privacy standards, transparency in data handling, and robust informed consent procedures for incidental findings. The interviewees advocate for realistic expectation management and public communication about precision medicine with biomarkers.

Further continuous empirical studies are needed to explore the perspectives of additional patient groups, their wishes and needs, including pregnant people as well as children, young and old people, that might be neglected in research. However, these are groups that are affected by AD/Pso [1, 126, 128], which, as chronic diseases, can span a whole life. These groups are often particularly vulnerable and might face specific challenges and needs (also for research). On the whole, diverse views representative of the populations for whom the biomarkers are developed are necessary to consider their needs and wishes. Furthermore, biomarker research itself should be based on diverse data representative for populations creating good evidence for fair access and more precise predictions, diagnostics and treatments.

Overall, striving for better treatment should not ignore the bigger picture of the lives of patients with AD/Pso and the wishes for human flourishing. The identification of ethical issues and the nuanced view of harm depicted in this article can help to weigh the risks and benefits of biomarker research and application in healthcare. This should inform policy development for data-rich biomarker use for AD/Pso as well as support research practice, public health interventions and clinical practice in developing and applying medical innovations ethically and socially responsibly. Sound harm mitigation strategies should support research on biomarkers and their application in such a way that harms are not only minimised but, more importantly, made visible and acknowledged.

## Supplementary Information

Supplementary Material 1.

## Data Availability

The datasets generated and analysed during the current study are not publicly available as whole interview transcripts cannot be completely de-identified. Legal restrictions apply to protect the identity of the participants as their consent was bound to confidentiality. The interview guide for the semi-structured interviews was made available to the editors and reviewers on request. The interview guide for the semi-structured interviews was made available to the editors and reviewers on request.

## Abbreviations

AD: Atopic Dermatitis
Pso: Psoriasis
AI: Artificial intelligence
ML: Machine learning

## Footnotes

1 More details on different definitions of biomarkers see Califf [20], and Strimbu and Tavel [21].

2 There is philosophical controversy over the use of event-based or state-based formulations for harm. Typically, counterfactual comparative accounts use a formulation based on “events”, see, e.g., Hanser [51], Rabenberg [52], Purves [53]. For formulations based on states, see e.g. Thomson [54] and Hanser [55].

3 Experiments have shown that communicating uncertainty in numbers had little effect regarding public trust [115]. The authors conclude that knowledge of this fact may enable science communicators and academics to increase transparency concerning the limitations of what humans can know [114].

## References

[1] Bylund S, V.K.L.B., Svalstedt M, Svensson Å, Prevalence and Incidence of Atopic Dermatitis: A Systematic Review. Acta DV Advances in dermatology and venereology, 2020. 100: adv00160. 10.2340/00015555-3510 PMC9189744 32412646

[2] Michalek IM, Loring B, John SM. A systematic review of worldwide epidemiology of psoriasis. J Eur Acad Dermatol Venereol. 2017;31(2):205–12. 27573025 10.1111/jdv.13854

[3] Parisi R, et al. National, regional, and worldwide epidemiology of psoriasis: systematic analysis and modelling study. BMJ. 2020;369: m1590. 32467098 10.1136/bmj.m1590 PMC7254147

[4] Griffiths CEM, Armstrong AW, Gudjonsson JE, Barker J. Psoriasis. Lancet. 2021;397(10281):1301–15. 33812489 10.1016/S0140-6736(20)32549-6

[5] Dauden E, et al. Defining well-being in psoriasis: A Delphi consensus among healthcare professionals and patients. Sci Rep. 2024;14(1):14519. 38914574 10.1038/s41598-024-64738-6 PMC11196587

[6] Langley RG, Krueger GG, Griffiths CE. Psoriasis: epidemiology, clinical features, and quality of life. Ann Rheum Dis. 2005;64(2):ii18-23. 10.1136/ard.2004.033217 PMC1766861 15708928

[7] Weidinger S, Beck LA, Bieber T, et al. Atopic dermatitis. Nat Rev Dis Primers. 2018;4:1-2. 10.1038/s41572-018-0001-z 29930242

[8] Sbidian E, Chaimani A, Garcia-Doval I, Doney L, Dressler C, Hua C, et al. Systemic pharmacological treatments for chronic plaque psoriasis: a network meta-analysis. Cochrane Database Syst Rev. 2022;5(5):CD011535. 10.1002/14651858.CD011535.pub5 PMC9125768 35603936

[9] Drucker AM, L.M., Prieto-Merino D, Malek R, Ellis AG, Yiu ZZN, Rochwerg B, Di Giorgio S, Arents BWM, Mohan T, Burton T, Spuls PI, Schmitt J, Flohr C. Systemic Immunomodulatory Treatments for Atopic Dermatitis: Living Systematic Review and Network Meta-Analysis Update. JAMA Dermatol. 2024;160(9):1012. 10.1001/jamadermatol.2024.2192 PMC11255974 39018058

[10] D’Angelo S, et al. Review of the treatment of psoriatic arthritis with biological agents: choice of drug for initial therapy and switch therapy for non-responders. Open Access Rheumatol. 2017;9:21–8. 28280401 10.2147/OARRR.S56073 PMC5338946

[11] Augustin M, Misery L, von Kobyletzki L, Armario-Hita JC, Mealing S, Redding M. , Unveiling the true costs and societal impacts of moderate-to-severe atopic dermatitis in Europe. J Eur Acad Dermatol Venereol, 2022.36(7):3-16. 10.1111/jdv.18168 35801296

[12] Avena-Woods C. Overview of atopic dermatitis. Am J Manag Care. 2017;23(8):S115–23. 28978208

[13] Bieber T, et al. Clinical phenotypes and endophenotypes of atopic dermatitis: Where are we, and where should we go? J Allergy Clin Immunol. 2017;139(4S):S58–64. 28390478 10.1016/j.jaci.2017.01.008

[14] Li Y, et al. Identification of novel immune subtypes and potential hub genes of patients with psoriasis. J Transl Med. 2023;21(1):182. 36890558 10.1186/s12967-023-03923-z PMC9993638

[15] Bakker D, et al. Biomarkers in atopic dermatitis. Journal of Allergy and Clinical Immunology. 2023;151(5):1163–8. 36792449 10.1016/j.jaci.2023.01.019

[16] Corbett M, et al. Biomarkers of systemic treatment response in people with psoriasis: a scoping review. Br J Dermatol. 2022;187(4):494–506. 35606928 10.1111/bjd.21677 PMC9796396

[17] Camela E, et al. Towards Personalized Medicine in Psoriasis: Current Progress. Psoriasis (Auckl). 2022;12:231–50. 36071793 10.2147/PTT.S328460 PMC9444142

[18] Yu L, Li L. Potential biomarkers of atopic dermatitis. Front Med (Lausanne). 2022;9:1028694. 36465933 10.3389/fmed.2022.1028694 PMC9712451

[19] FDA-NIH Biomarker Working Group. BEST (biomarkers, EndpointS, and other tools) Recource [internet]. Silver Spring (MD): Food and Drug Administration (US); 2016. https://www.ncbi.nlm.nih.gov/books/NBK338448/ (last accessed 04 August 2025) Co-published by National Institutes of Health (US), Bethesda (MD).

[20] Califf RM. Biomarker definitions and their applications. Exp Biol Med (Maywood). 2018;243(3):213–21. 29405771 10.1177/1535370217750088 PMC5813875

[21] Strimbu K, Tavel JA. What are biomarkers? Curr Opin HIV AIDS. 2010;5(6):463–6. 20978388 10.1097/COH.0b013e32833ed177 PMC3078627

[22] Kawano-Dourado L, et al. Proactive therapeutic drug monitoring of biologic drugs in adult patients with inflammatory bowel disease, inflammatory arthritis, or psoriasis: a clinical practice guideline. BMJ. 2024;387: e079830. 39467592 10.1136/bmj-2024-079830

[23] Mortlock RD, Ma EC, Cohen JM, Damsky W. Assessment of Treatment-Relevant Immune Biomarkers in Psoriasis and Atopic Dermatitis: Toward Personalized Medicine in Dermatology. J Invest Dermatol. 2023;143(8):1412–22. 37341663 10.1016/j.jid.2023.04.005 PMC10830170

[24] Broderick C, et al. Biomarkers associated with the development of comorbidities in patients with atopic dermatitis: A systematic review. Allergy. 2023;78(1):84–120. 36366871 10.1111/all.15578 PMC10107168

[25] Broderick C, et al. The BIOMarkers in Atopic Dermatitis and Psoriasis (BIOMAP) glossary: developing a lingua franca to facilitate data harmonization and cross‐cohort analyses. Br J Dermatol. 2021;185(5):1066–1069. 10.1111/bjd.20587 34137018

[26] Ramessur R, et al. Defining disease severity in atopic dermatitis and psoriasis for the application to biomarker research: an interdisciplinary perspective. Br J Dermatol. 2024;191(1):14-23.. 10.1093/bjd/ljae080 PMC11188926 38419411

[27] Ramessur R, Corbett M, Marshall D, Acencio ML, Barbosa IA, Dand N, et al. BIOMAP consortium. Biomarkers of disease progression in people with psoriasis: a scoping review. Br J Dermatol. 2022;187(4):481-93. 10.1111/bjd.21627 PMC9796834 35482474

[28] Ziehfreund S, et al. Requirements and expectations of high-quality biomarkers for atopic dermatitis and psoriasis in 2021-a two-round Delphi survey among international experts. J Eur Acad Dermatol Venereol. 2022;36(9):1467-1476. 10.1111/jdv.18178 35470457

[29] Ring J, et al. Atopic eczema: burden of disease and individual suffering - results from a large EU study in adults. J Eur Acad Dermatol Venereol. 2019;33(7):1331–40. 31002197 10.1111/jdv.15634

[30] Gore C, et al. The information needs and preferred roles in treatment decision-making of parents caring for infants with atopic dermatitis: a qualitative study. Allergy. 2005;60(7):938–43. 15932385 10.1111/j.1398-9995.2005.00776.x

[31] Baum ML. The Neuroethics of Biomarkers: What the Development of Bioprediction Means for Moral Responsibility, Justice, and the Nature of Mental Disorder. New York, NY: Oxford University Press; 2016.

[32] Smedinga M, Tromp K, Schermer MHN, Richard E. Ethical Arguments Concerning the Use of Alzheimer's Disease Biomarkers in Individuals with No or Mild Cognitive Impairment: A Systematic Review and Framework for Discussion. J Alzheimers Dis. 2018;66(4):1309-1322. 10.3233/JAD-180638 30507575

[33] Blanchard A. Mapping ethical and social aspects of cancer biomarkers. New Biotechnol. 2016;33(6):763-772. 10.1016/j.nbt.2016.06.1458 27318010

[34] Fritzsche MC, Buyx AM, Hangel N. Mapping ethical and social aspects of biomarker research and its application in atopic dermatitis and psoriasis: a systematic review of reason. J Eur Acad Dermatol Venereol. 2022;36(8):1201-1213.. 10.1111/jdv.18128 35366351

[35] Döringer S, ‘The problem-centred expert interview’. Combining qualitative interviewing approaches for investigating implicit expert knowledge. Int J Soc Res Methodol. 2020; 24(3):265–278.

[36] Molewijk B, Stiggelbout AM, Otten W, Dupuis HM & Kievit J, Empirical data and moral theory. A plea for integrated empirical ethics. Med Health Care Philosophy. 2004; 7(1): p. 55–69. 10.1023/b:mhep.0000021848.75590.b0 15139255

[37] Beauchamp TL, Childress JF, Principles of biomedical ethics. Eighth edition ed. 2019, New York, N.Y. Oxford: Oxford Univ. Press.

[38] Zhou YK. What it means to suffer harm. Jurisprudence. 2021;13(1):26–51.

[39] Fricker M, Epistemic injustice: power and the ethics of knowing. Vol. 69. 2007. New York: Oxford University Press. 380–382.

[40] Hanser M. Understanding Harm and its Moral Significance. Ethical Theory Moral Pract. 2019;22(4):853–70.

[41] Mill JS, On liberty / Über die Freiheit: englisch - deutsch. Ed. B. Gräfrath. Vol. 18536. Stuttgart: Reclam 2009.

[42] Bradley B. Doing Away with Harm1. Philos Phenomenol Res. 2012;85(2):390–412.

[43] Shiffrin SV. Harm and Its Moral Significance. Leg Theory. 2012;18(3):357–98.

[44] Gardner M, What Is Harming?, in Principles and Persons: The Legacy of Derek Parfit, J. McMahan, T. Campbell, J. Goodrich, and K. Ramakrishnan, Editors. 2021. Oxford: Oxford University Press.

[45] Tadros V, Wrongs and crimes. 2016. Oxford: Oxford University Press.

[46] Petersen TS. Being Worse Off: But in Comparison with What? On the Baseline Problem of Harm and the Harm Principle. Res Publica. 2014;20(2):199–214.

[47] Purshouse C. A Defence of the Counterfactual Account of Harm. Bioethics. 2016;30(4):251–9. 26423790 10.1111/bioe.12207

[48] Feinberg J, The moral limits of the criminal law. Vol. 15. 1984. New York: Oxford University Press. 381–395.

[49] Norcross A. Harming In Context. Philos Stud. 2005;123(1–2):149–73.

[50] Foddy B. In Defense of a Temporal Account of Harm and Benefit. American Philosophical Quarterly. 2014;51(2):155–65.

[51] Hanser M. The Metaphysics of Harm. Philos Phenomenol Res. 2008;77(2):421–50.

[52] Rabenberg, M., Harm. Journal of Ethics and Social Philosophy, 2015. 8(3): p. 1–32.

[53] Purves D. Harming as making worse off. Philos Stud. 2018;176(10):2629–56.

[54] Thomson JJ. More On The Metaphysics of Harm. Philos Phenomenol Res. 2010;82(2):436–58.

[55] Hanser M. Still More on the Metaphysics of Harm. Philos Phenomenol Res. 2011;82(2):459–69.

[56] Unruh CF. A Hybrid Account of Harm. Austrl J Philosophy. 2022.101 (4):890-903.

[57] Bozzaro C. Der Leidensbegriff im medizinischen Kontext: Ein Problemaufriss am Beispiel der tiefen palliativen Sedierung am Lebensende. Ethik in der Medizin. 2015;27(2):93–106.

[58] Maio, G., C. Bozzaro, and T. Eichinger, Leid und Schmerz: konzeptionelle Annäherungen und medizinethische Implikationen. 2015: Verlag Karl Alber.

[59] Bendelow GA, Williams SJ. The Lived Body: Sociological Themes, Embodied Issues. Routledge; 1998.

[60] Leder D, The Absent Body. 1990. Chicago: University of Chicago Press.

[61] Svenaeus F. The phenomenology of suffering in medicine and bioethics. Theor Med Bioeth. 2014;35(6):407–20. 25398688 10.1007/s11017-014-9315-3

[62] Tong A, Sainsbury P, Craig J. Consolidated criteria for reporting qualitative research (COREQ): a 32-item checklist for interviews and focus groups. Int J Qual inHealth Care. 2007;19(6):349–57. 10.1093/intqhc/mzm042 17872937

[63] Charmaz, K., Constructing grounded theory. 2nd edition ed. Introducing qualitative methods. 2014, London ; Thousand Oaks, Calif.: Sage. xxi, 388 pages.

[64] Bryman A, Social Research Methods. 5th edition ed. 2015. Oxford: Oxford University Press.

[65] Yin R. Qualitative research from start to finish. Second edition. ed. 2016, New York: Guilford Press. xxix, 386 pages.

[66] Hangel N, Buyx A, Fritzsche MC. The interrelation of scientific, ethical, and translational challenges for precision medicine with multimodal biomarkers - A qualitative expert interview study in dermatology research. Heliyon. 2024;10(13):e31723. 10.1016/j.heliyon.2024.e31723 PMC11260963 39040296

[67] McLennan S, Fiske A, Celi LA, et al. An embedded ethics approach for AI development. Nat Mach Intell. 2020;2:488–490.

[68] Tigard DW, Braun M, Breuer S, Ritt K, Fiske A, McLennan S, et al. Toward best practices in embedded ethics: Suggestions for interdisciplinary technology development. Robot Auton Syst. 2023;167:104467.

[69] Willem T, Fritzsche MC, Zimmermann BM, et al. Embedded Ethics in Practice: A Toolbox for Integrating the Analysis of Ethical and Social Issues into Healthcare AI Research. Sci Eng Ethics. 2025;31:3. 10.1007/s11948-024-00523-y PMC11668859 39718728

[70] Schleidgen S, et al. What is personalized medicine: sharpening a vague term based on a systematic literature review. BMC Med Ethics. 2013;14:55. 24359531 10.1186/1472-6939-14-55 PMC3878093

[71] Juengst E, McGowan ML, Fishman JR, Settersten RA Jr. From “Personalized” to “Precision” Medicine: The Ethical and Social Implications of Rhetorical Reform in Genomic Medicine. Hastings Cent Rep. 2016;46(5):21–33. 27649826 10.1002/hast.614 PMC5153661

[72] Wang RS, Maron BA, Loscalzo J. Systems medicine: evolution of systems biology from bench to bedside. Wiley Interdiscip Rev Syst Biol Med. 2015;7(4):141–61. 25891169 10.1002/wsbm.1297 PMC4457580

[73] Green S, Prainsack B, Sabatello M. The roots of (in)equity in precision medicine: gaps in the discourse. Per Med. 2024;21(1):5–9. 38088178 10.2217/pme-2023-0097 PMC10784620

[74] Németh B, Csanádi M, Inotai A, Ameyaw D, Káló Z, Access to high-priced medicines in lower-income countries in the WHO European. Oslo Medicines Initiative Technical Report World Health Organisation 2022. 36542735

[75] Green S, Vogt H. Personalizing medicine in silico and in socio. Humana Mente Journal of Philosophical Studies. 2016;9(30):105–45.

[76] Quattrocchi A, et al. Personalized medicine in psychiatric disorders: prevention and bioethical questions. Clin Ter. 2019;170(6):e421–4. 31696903 10.7417/CT.2019.2169

[77] Meunier R, Herzog C. Omics and AI in precision medicine: Maintaining socio-technical imaginaries by transforming technological assemblages. TATuP - Zeitschrift für Technikfolgenabschätzung in Theorie und Praxis. 2023;32(3):48–53.

[78] Smart A, M.P., Parker M. , Tailored medicine: whom will it fit? The ethics of patient and disease stratification. . Bioethics, 2004 Aug. 18(4): p. 322–42. 10.1111/j.1467-8519.2004.00400.x 15449405

[79] Vayena E, Blasimme A, Cohen IG. Machine learning in medicine: Addressing ethical challenges. PLoS Med. 2018;15(11): e1002689. 30399149 10.1371/journal.pmed.1002689 PMC6219763

[80] Vayena E, Salathé M, Madoff LC, Brownstein JS. Ethical challenges of big data in public health. PLoS Comput Biol. 2015;11(2): e1003904. 25664461 10.1371/journal.pcbi.1003904 PMC4321985

[81] Obermeyer Z, Emanuel EJ. Predicting the Future - Big Data, Machine Learning, and Clinical Medicine. N Engl J Med. 2016;375(13):1216–9. 27682033 10.1056/NEJMp1606181 PMC5070532

[82] Savulescu J, Giubilini A, Vandersluis R, Mishra A. Ethics of artificial intelligence in medicine. Singapore Med J. 2024;65(3):150–8. 38527299 10.4103/singaporemedj.SMJ-2023-279 PMC7615805

[83] Char DS, Shah NH, Magnus D. Implementing Machine Learning in Health Care - Addressing Ethical Challenges. N Engl J Med. 2018;378(11):981–3. 29539284 10.1056/NEJMp1714229 PMC5962261

[84] Federico CA, Trotsyuk AA. Biomedical Data Science, Artificial Intelligence, and Ethics: Navigating Challenges in the Face of Explosive Growth. Annu Rev Biomed Data Sci. 2024;7(1):1–14. 38598860 10.1146/annurev-biodatasci-102623-104553

[85] Chung J, Simpson EL. The socioeconomics of atopic dermatitis. Ann Allergy Asthma Immunol. 2019;122(4):360–6. 30597208 10.1016/j.anai.2018.12.017

[86] Drucker AM, et al. The Burden of Atopic Dermatitis: Summary of a Report for the National Eczema Association. J Invest Dermatol. 2017;137(1):26–30. 27616422 10.1016/j.jid.2016.07.012

[87] WHO, Global report on psoriasis. 2016: WHO Press, Geneva (Switzerland).

[88] Marron SE, et al. The psychosocial burden of hand eczema: Data from a European dermatological multicentre study. Contact Dermatitis. 2018;78(6):406–12. 29464713 10.1111/cod.12973

[89] Haugeberg G, et al. Physical and Psychosocial Burden of Psoriatic Arthritis: Longitudinal Data From a Population-Based Study in Norway. Arthritis Care Res (Hoboken). 2021;73(1):138–45. 33242358 10.1002/acr.24412

[90] Kimball AB, J.C., Weiss S, Vreeland MG, Wu Y. The psychosocial burden of psoriasis. Am J Clin Dermatol. 2005; 6(6): p. 383–92. 10.2165/00128071-200506060-00005 16343026

[91] Santer M, et al. Experiences of carers managing childhood eczema and their views on its treatment: a qualitative study. Br J Gen Pract. 2012;62(597):e261–7. 22520913 10.3399/bjgp12X636083 PMC3310032

[92] Howells LM, et al. “When it goes back to my normal I suppose”: a qualitative study using online focus groups to explore perceptions of “control” among people with eczema and parents of children with eczema in the UK. BMJ Open. 2017;7(11): e017731. 10.1136/bmjopen-2017-017731 PMC5695402 29146642

[93] Boonin D. The Non-Identity Problem and the Ethics of Future People. 2014. Oxford: Oxford University Press.

[94] Gardner M. A Harm-Based Solution to the Non-Identity Problem. Ergo, an Open Access J Philos. 2015;2(20201214).

[95] Bueno-Gomez N. Conceptualizing suffering and pain. Philos Ethics Humanit Med. 2017;12(1):7. 28958214 10.1186/s13010-017-0049-5 PMC5621131

[96] Throop CJ. Experience, Coherence, and Culture: The Significance of Dilthey’s “Descriptive Psychology” for the Anthropology of Consciousness. Anthropol Conscious. 2002;13(1):2–26.

[97] Dilthey W. Der Aufbau der Geschichtlichen Welt in den Geisteswissenschaften [The Construction of the Historical World in the Human Studies]. 2013, Berlin: Holzinger.

[98] Crenshaw K. Demarginalizing the Intersection of Race and Sex: A Black Feminist Critique of Antidiscrimination Doctrine, Feminist Theory and Antiracist Politics. The University of Chicago Legal Forum. 1989;140:139–67.

[99] Collins HP. Intersectionality as critical social theory. Durham and London: Duke University Press; 2019.

[100] Collins PH and Bilge S. Intersectionality. 2020. Cambridge: Polity.

[101] Sirugo G, Williams SM, Tishkoff SA. The Missing Diversity in Human Genetic Studies. Cell. 2019;177(1):26–31. 30901543 10.1016/j.cell.2019.02.048 PMC7380073

[102] Bentley AR, Callier SL, Rotimi CN. Evaluating the promise of inclusion of African ancestry populations in genomics. NPJ Genom Med. 2020;5:5. 32140257 10.1038/s41525-019-0111-x PMC7042246

[103] Malinowska JK, Zuradzki T. Reductionist methodology and the ambiguity of the categories of race and ethnicity in biomedical research: an exploratory study of recent evidence. Med Health Care Philos. 2023;26(1):55–68. 36352325 10.1007/s11019-022-10122-y PMC9646278

[104] Crichton P, Carel H, Kidd IJ. Epistemic injustice in psychiatry. BJPsych Bull. 2017;41(2):65–70. 28400962 10.1192/pb.bp.115.050682 PMC5376720

[105] Carel H, Kidd IJ. Epistemic injustice in healthcare: a philosophial analysis. Med Health Care Philos. 2014;17(4):529–40. 24740808 10.1007/s11019-014-9560-2

[106] Aristoteles, H.G., Die Nikomachische Ethik: Griechisch - Deutsch. 2007: De Gruyter.

[107] Nussbaum, M.C., Creating Capabilities: The Human Development Approach. 2011: Harvard University Press.

[108] Collingridge, D., The Social Control of Technology. 1980. New York: St. Martin's Press.

[109] Borup M, Brown N, Konrad K, Van Lente H. The sociology of expectations in science and technology. Technol Anal Strateg Manag. 2006;18(3–4):285–98.

[110] Brown N, Michael M, A sociology of expectations: Retrospecting prospects and prospecting retrospects. Technology Analysis & Strategic Management, 2003. 15(1): p. 3–18. a

[111] Caulfield T. Biotechnology and the popular press: hype and the selling of science. Trends Biotechnol. 2004;22(7):337–9. 15245905 10.1016/j.tibtech.2004.03.014

[112] Strange M. Three different types of AI hype in healthcare. AI and Ethics. 2024;4(3):833–40.

[113] Emanuel EJ, Wachter RM. Artificial Intelligence in Health Care: Will the Value Match the Hype? JAMA. 2019;321(23):2281–2. 31107500 10.1001/jama.2019.4914

[114] Ameen M, et al. Perception and Experience of Biologic Therapy in Atopic Dermatitis: A Qualitative Focus Group Study of Physicians and Patients in Europe and Canada. Dermatol Ther (Heidelb). 2021;11(6):2159–77. 34704230 10.1007/s13555-021-00631-8 PMC8547298

[115] van der Bles AM, van der Linden S, Freeman ALJ, Spiegelhalter DJ. The effects of communicating uncertainty on public trust in facts and numbers. Proc Natl Acad Sci U S A. 2020;117(14):7672–83. 32205438 10.1073/pnas.1913678117 PMC7149229

[116] Lucivero F, Swierstra T, Boenink M. Assessing Expectations: Towards a Toolbox for an Ethics of Emerging Technologies. NanoEthics. 2011;5(2):129–41. 21957435 10.1007/s11569-011-0119-x PMC3166601

[117] Deyo RA, Patrick DL. Hope or hype the obsession with medical advances and the high cost of false promises. New York: AMACOM, American Management Association; 2005.

[118] van Lente H. Promising technology: the dynamics of expectations in technological developments. Universiteit Twente, Faculteit Wijsbegeerte en Maatschappijwetenschappen; 1993.

[119] Pona A. et al., The ethical foundation for honesty and the focused use of deception in dermatology. Dermatol Online J. 2020; 26(11). 33342174

[120] Floridi L, et al. AI4People-An Ethical Framework for a Good AI Society: Opportunities, Risks, Principles, and Recommendations. Minds Mach (Dordr). 2018;28(4):689–707. 30930541 10.1007/s11023-018-9482-5 PMC6404626

[121] Adadi A, Berrada M. Peeking Inside the Black-Box: A Survey on Explainable Artificial Intelligence (XAI). IEEE Access. 2018;6:52138–60.

[122] Vilone G, Longo L . Explainable artificial intelligence: a systematic review. arXiv preprint https://arxiv.org/abs/2006.00093, 2020.

[123] Arbelaez Ossa L, et al. Re-focusing explainability in medicine. Digit. Health. 2022;8:20552076221074490. 10.1177/20552076221074488 PMC8841907 35173981

[124] Lekadir K, et al. FUTURE-AI: international consensus guideline for trustworthy and deployable artificial intelligence in healthcare. BMJ. 2025;388: e081554. 39909534 10.1136/bmj-2024-081554 PMC11795397

[125] Goisauf M, Cano Abadía M, Akyüz K, Bobowicz M, Buyx A, Colussi I, Fritzsche MC, Lekadir K, Marttinen P, Mayrhofer MT, Meszaros J. Trust, Trustworthiness, and the Future of Medical AI: Outcomes of an Interdisciplinary Expert Workshop. J Med Internet Res 2025;27:e71236 10.2196/71236 PMC12171647 40455564

[126] Balakirski G, Novak N. Atopic dermatitis and pregnancy. J Allergy Clin Immunol. 2022;149(4):1185–94. 35090948 10.1016/j.jaci.2022.01.010

[127] Roest J, Nkosi B, Seeley J, Molyneux S, Kelley M. Respecting relational agency in the context of vulnerability: What can research ethics learn from the social sciences? Bioethics. 2023;37(4):379–88. 36709500 10.1111/bioe.13139 PMC10946974

[128] Abuabara K, et al. The prevalence of atopic dermatitis beyond childhood: A systematic review and meta-analysis of longitudinal studies. Allergy. 2018;73(3):696–704. 28960336 10.1111/all.13320 PMC5830308

