# Supplementary figures and images for: Ethical challenges in biomarker research and precision medicine – a qualitative study in dermatology
Source: BMC Med Ethics. 2025 Nov 18;26:162. doi: 10.1186/s12910-025-01258-6 (PMC12625220; doi:10.1186/s12910-025-01258-6)

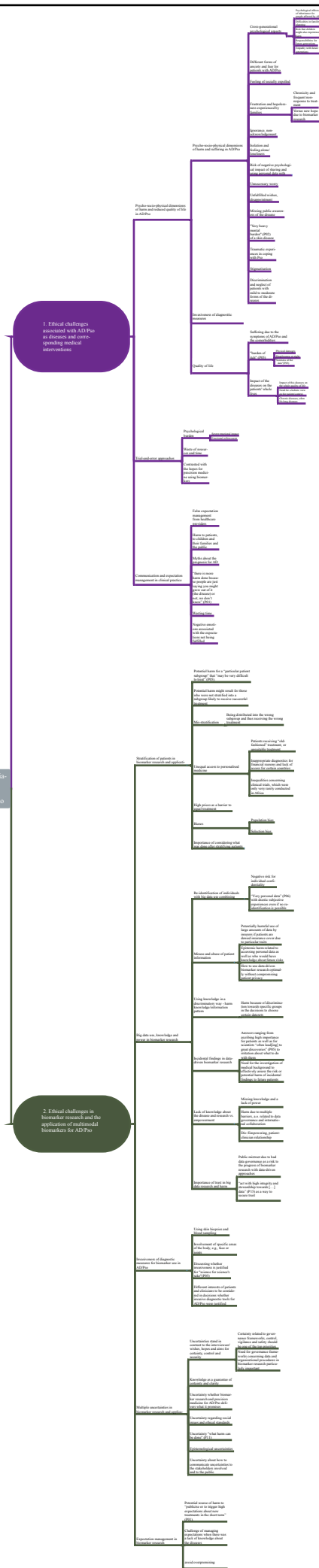

Supplement: Supplementary file 1 — Supplementary Material 1. [file 12910_2025_1258_MOESM1_ESM.pdf]
